# Vasospastic Angina Unmasked on Repeat Coronary Angiography in a Patient Without Typical Triggers: A Case Report

**DOI:** 10.7759/cureus.81139

**Published:** 2025-03-25

**Authors:** Khaleel Quasem, Michelle Carrasquel, Dania Baraka

**Affiliations:** 1 Internal Medicine, McLaren Greater Lansing, Lansing, USA

**Keywords:** angina, cardiology, coronary artery disease, prinzmental angina, vasospastic angina

## Abstract

Vasospastic angina (Prinzmetal angina) is a rare but clinically significant cause of chest pain that occurs due to transient coronary artery spasm, leading to myocardial ischemia. It often presents with chest pain at rest, typically during late-night or early-morning hours, and may be associated with transient ST-segment changes on an electrocardiogram (ECG) but without significant obstructive coronary disease. Typical triggers include cigarette smoking, drug use, and stress-related endothelial dysfunction. However, some patients lack traditional risk factors, complicating diagnosis. We describe a 69-year-old woman with a history of ST-elevation myocardial infarction (STEMI), hypertension, hyperlipidemia, and venous thromboembolism, who presented with left-sided chest pain, nausea, and diaphoresis. Initial workup suggested a non-ST-elevation myocardial infarction (NSTEMI) with elevated cardiac troponin, yet urgent coronary angiography showed no obstructive lesions. Persistent symptoms and further troponin elevation (>2000 ng/L) prompted a second angiography, which revealed coronary vasospasm in the proximal left anterior descending (LAD) and circumflex arteries, confirming NSTEMI precipitated by intense, prolonged vasospasm. The patient responded well to calcium channel blockers and long-acting nitrates, underscoring the need to consider vasospastic angina even in individuals without common precipitants. This case highlights the importance of repeat angiography, the role of vasodilatory therapy, and the potential for more advanced diagnostic or genetic testing to clarify underlying risk factors.

## Introduction

Vasospastic angina was first described in 1959 by Prinzmetal et al. as a “variant form of angina pectoris,” featuring transient coronary artery spasm leading to myocardial ischemia and characteristic ST-segment elevation on an electrocardiogram (ECG) [1,2]. In contrast to angina pectoris primarily caused by fixed atherosclerotic stenoses, episodes of vasospastic angina often occur at rest, particularly from late night to early morning, and may present with pronounced ST-segment elevation or other transient ECG changes [3-5]. The most reliable risk factor identified is cigarette smoking; however, illicit drugs such as cocaine can also precipitate coronary spasm [2,3]. While risk factors exist, vasospastic angina can still occur in the absence of these factors, complicating the diagnosis [4].

The exact pathophysiology is not fully understood, but endothelial dysfunction, hyperreactivity of vascular smooth muscle cells, and autonomic nervous system imbalances have been implicated [6]. Genetic predisposition (e.g., variants in endothelial nitric oxide synthase (eNOS)) may also increase susceptibility [7]. Coronary angiography is crucial for diagnosis, demonstrating the transient nature of spasm in the absence of obstructive lesions, sometimes using provocative testing with ergonovine or acetylcholine [5,8]. However, false negatives may occur when vasospasm is intermittent or if the underlying trigger is not present at the time of testing [8]. Noninvasive modalities, including ambulatory ECG monitoring, MRI, and PET, may detect transient ischemia but remain adjunctive to invasive assessment [9,10].

Untreated or underrecognized vasospastic angina can lead to serious outcomes, including significant myocardial infarction (as in this case), life-threatening arrhythmias, and sudden cardiac death [2,6]. Even in patients with a history of atherosclerotic disease (e.g., ST-elevation myocardial infarction (STEMI)) or other cardiovascular comorbidities, vasospasm may arise without typical precipitants like smoking or drug use, thus complicating the clinical picture. A high index of suspicion is therefore essential to ensure prompt diagnosis and appropriate therapy.

## Case presentation

A 69-year-old woman presented with sudden-onset left-sided substernal chest pain, accompanied by nausea and diaphoresis. She had a history of STEMI, hypertension, hyperlipidemia, recent deep vein thrombosis, and saddle pulmonary embolism (status post-thrombectomy) on rivaroxaban. She did not smoke, consume alcohol, or use illicit drugs, and there were no new stressors or changes in medication. Her vital signs were stable, and her blood pressure remained within normal limits (Table 1).

**Table 1:** Summary of case presentation. This table provides a comprehensive summary of the patient's demographic details, past medical history, presenting symptoms, diagnostic findings, and treatment course. DVT: deep vein thrombosis; ECG: electrocardiogram; LVEF: left ventricular ejection fraction; LAD: left anterior descending.

Parameter	Details
Age	69 years
Gender	Female
Past medical history	STEMI, hypertension, hyperlipidemia, recent DVT, saddle pulmonary embolism (post-thrombectomy)
Current medications	Rivaroxaban, nebivolol, amlodipine, isosorbide mononitrate
Presenting symptoms	Left-sided substernal chest pain, nausea, diaphoresis
Initial troponin level	21 ng/L, rising to 110 ng/L
ECG findings	No ST-segment elevations, no significant ischemic changes
First coronary angiography	No significant coronary artery disease, preserved LVEF
Troponin peak	>2000 ng/L
Second coronary angiography	Coronary vasospasm in proximal LAD and circumflex arteries
Final diagnosis	Vasospastic angina
Discharge medications	Nebivolol 10 mg daily, amlodipine 5 mg twice daily, isosorbide mononitrate 30 mg daily
Follow-up plan	Cardiology follow-up, medication titration, lifestyle modifications

Upon admission, her high-sensitivity troponin level was 21 ng/L (reference <15 ng/L), which then rose to 110 ng/L. Serial ECGs did not show classic ST-segment elevation or depression; however, transient T-wave inversions were noted in the lateral leads during one episode of chest pain. With ongoing chest pain and elevated troponin levels, she was diagnosed with a non-ST-elevation myocardial infarction (NSTEMI). Urgent coronary angiography revealed patent coronary arteries with normal flow and preserved left ventricular ejection fraction. Despite the absence of obstructive lesions, her chest pain continued, and troponin levels rose further to >2000 ng/L within 24 hours. A repeat limited echocardiogram showed no new wall motion abnormalities (Table 2).

**Table 2:** Laboratory data on admission. These laboratory findings support the diagnosis of NSTEMI, with an elevated high-sensitivity troponin level, and otherwise unremarkable inflammatory and metabolic parameters. NSTEMI: non-ST elevation myocardial infarction; LDL: low-density lipoprotein; HDL: high-density lipoprotein; BNP: B-type natriuretic peptide.

Parameter	Admission Value	Peak Value	Reference Range
Hemoglobin (g/dL)	13.2	13.2	12.0-16.0
White blood cell count (10^3^/µL)	7.8	7.8	4.0-11.0
Platelet count (10^3^/µL)	240	240	150-400
Sodium (mmol/L)	138	138	135-145
Potassium (mmol/L)	4.2	4.2	3.5-5.1
Creatinine (mg/dL)	0.9	0.9	0.6-1.3
High-sensitivity troponin I (ng/L)	21	110	<15
Total cholesterol (mg/dL)	180	180	<200
LDL cholesterol (mg/dL)	110	110	<100
HDL cholesterol (mg/dL)	45	45	>40
Triglycerides (mg/dL)	120	120	<150
C-reactive protein (mg/L)	2.5	2.5	<3.0
BNP (pg/mL)	85	85	<100
D-dimer (ng/mL)	280	280	<500

**Figure 1:**
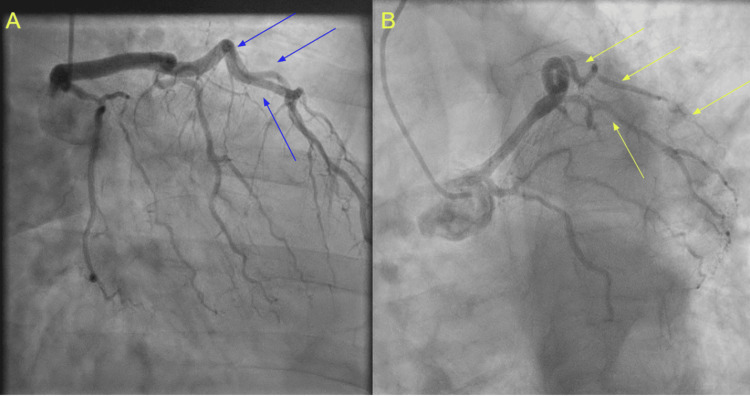
Coronary angiography demonstrating vasospastic angina. (A) Initial angiography demonstrating normal coronary arteries without significant stenosis (blue arrows). (B) Repeat angiography showing severe vasospasm in the LAD and left circumflex arteries (yellow arrows), leading to critical narrowing. These findings confirm dynamic coronary vasospasm as the underlying etiology of the patient's symptoms. LAD: left anterior descending.

Suspecting an alternative mechanism of myocardial injury, a second angiography was performed two days later to evaluate for dynamic changes. This procedure demonstrated severe coronary vasospasm in the proximal LAD and left circumflex arteries (Figure 1). Intracoronary nitroglycerin promptly relieved the spasm and restored normal caliber, confirming the diagnosis of coronary vasospasm as the cause of her NSTEMI. She remained hemodynamically stable throughout, with no documented arrhythmias. She was ultimately diagnosed with vasospastic angina, an NSTEMI secondary to coronary artery spasm, and discharged on nebivolol 10 mg daily, amlodipine 5 mg twice daily, and isosorbide mononitrate 30 mg once daily.

Upon discharge, the patient was counseled to avoid potential vasospastic triggers, such as extreme cold exposure and undue stress, and advised to continue her medications. A close outpatient follow-up was scheduled, during which ambulatory ECG monitoring would be considered if recurrent symptoms arose. At her two-week follow-up visit, she reported a significant improvement in both the frequency and severity of her chest pain.

## Discussion

This case underscores that intense or prolonged coronary artery spasm can precipitate clinically significant myocardial injury (NSTEMI), even in patients without hallmark triggers such as smoking or cocaine use [2]. Although “Prinzmetal angina” often evokes a picture of transient ST-segment elevation and minimal troponin release, real-world presentations span from brief, mild episodes to severe ischemic events comparable to those caused by plaque rupture [3,9]. The pathophysiological basis involves heightened vascular smooth muscle reactivity, endothelial dysfunction, and, in certain individuals, genetic polymorphisms (e.g., in eNOS), all of which may facilitate vasospasm in the absence of external precipitants [4-6]. Autonomic dysregulation, particularly increased vagal tone at night, can further predispose susceptible patients to coronary spasm. Although typical risk factors (e.g., cigarette smoking, drug use) were missing here, the patient’s history of coronary disease (prior STEMI) and hyperlipidemia likely contributed to endothelial dysfunction, thus lowering the threshold for vasospastic episodes.

Initial angiography was nondiagnostic despite marked troponin elevation, prompting a high index of suspicion and a repeat procedure once symptoms and biomarkers remained incongruent with normal coronary findings [7]. This second look revealed dynamic vasospasm readily reversed by intracoronary nitroglycerin. Had vasospasm not been visualized spontaneously, provocative testing (ergonovine, acetylcholine) could have confirmed the diagnosis, although it carries the risk of severe or prolonged spasm [8]. Noninvasive imaging modalities, such as ambulatory ECG monitoring or cardiac MRI-based stress perfusion, may also detect transient ischemia when invasive studies remain inconclusive. Management centers on long-acting nitrates and calcium-channel blockers, which reduce vasoconstriction and recurrent ischemic episodes [2,9]. While nonselective beta-blockers can worsen coronary spasm, nebivolol, given its β1 selectivity and vasodilatory properties, can be an acceptable choice for blood pressure control in patients with vasospastic angina and a history of myocardial infarction [10]. In refractory cases, nicorandil (a K⁺ channel opener and nitric oxide donor) or fasudil (a Rho-kinase inhibitor) may provide additional benefit. Close outpatient follow-up is essential to monitor for recurrent angina or arrhythmias, and further tests may be necessary to rule out microvascular dysfunction or anatomical variants if symptoms persist. Endothelial function assessments and genetic analyses could, in turn, refine risk stratification in patients who lack the classic profile for vasospastic angina.

## Conclusions

This case demonstrates that vasospastic angina can progress to NSTEMI in the absence of typical external triggers. Persistent symptoms and escalating troponin after a negative initial angiogram warranted a second look, which revealed dynamic coronary spasm. Early recognition and vasodilatory therapy (calcium channel blockers and long-acting nitrates) proved effective, and short-term follow-up showed significant clinical improvement. Future investigations, including genetic analyses, endothelial function testing, and prospective registries, may further elucidate individualized risk profiles and optimize management for vasospastic angina in diverse patient populations.
